# Effects of pay-for-performance based antimicrobial stewardship on antimicrobial consumption and expenditure: An interrupted time series analysis

**DOI:** 10.1016/j.heliyon.2024.e32750

**Published:** 2024-06-08

**Authors:** Haohai Xia, Jia Li, Xinyi Yang, Yingchao Zeng, Lin Shi, Weibin Li, Xu Liu, Shifang Yang, Manzhi Zhao, Jie Chen, Lianping Yang

**Affiliations:** a School of Public Health, Sun Yat-sen University, Guangzhou, China; b Department of Pharmacy, The First Affiliated Hospital, Sun Yat-sen University, Guangzhou, China; c Department of Infectious Disease, The Fifth Affiliated Hospital, Sun Yat-sen University, Zhuhai, China; d Department of Pulmonary and Critical Care Medicine, Guangdong Provincial People's Hospital, Guangdong Academy of Medical Sciences, Southern Medical University, Guangzhou, China

**Keywords:** Antimicrobial stewardship, One-off bonus payment, Penalty, Antimicrobial consumption, Interrupted time series analysis

## Abstract

**Objectives:**

To evaluate the impact of pay-for-performance on antimicrobial consumption and antimicrobial expenditure in a large teaching hospital in Guangzhou, China.

**Methods:**

We collected data from hospital information system from January 2018 through September 2022 in the inpatient wards. Antimicrobial consumption was evaluated using antibiotic use density (AUD) and antibiotic use rate (AUR). The economic impact of intervention was assessed by antimicrobial expenditure percentage. The data was analyzed using interrupted time series (ITS) analysis.

**Results:**

Following the implementation of the intervention, immediate decreases in the level of AUD were observed in Department of Hematology Unit 3 (β = −66.93 DDDs/100PD, *P* = 0.002), Urology (β = −32.80 DDDs/100PD, *P* < 0.001), Gastrointestinal Surgery Unit 3 (β = −11.44 DDDs/100PD, *P* = 0.03), Cardiac Surgery (β = −14.30 DDDs/100PD, *P* = 0.01), ICU, Unit 2 (β = −81.91 DDDs/100PD, *P* = 0.02) and Cardiothoracic Surgery ICU (β = −41.52 DDDs/100PD, *P* = 0.05). Long-term downward trends in AUD were also identified in Organ Transplant Unit (β = −1.64 DDDs/100PD, *P* = 0.02). However, only Urology (β = −6.56 DDDs/100PD, *P* = 0.02) and Gastrointestinal Surgery Unit 3 (β = −8.50 %, *P* = 0.01) showed an immediate decrease in AUR, and long-term downward trends in AUR were observed in Pediatric ICU (β = −1.88 %, *P* = 0.05) and ICU Unit 1 (β = −0.55 %, *P* = 0.02).

**Conclusion:**

This study demonstrates that the adoption of pay-for-performance effectively reduces antibiotic consumption in specific departments of a hospital in Guangzhou in the short term. However, it is important to recognize that the long-term impact of such interventions is often limited. Additionally, it should be noted that the overall effectiveness of the intervention across the entire hospital was not significant.

## 1 Introduction

The discovery of antibiotics stands out as one of the most significant medical advancements of the 20th century [1]. Regrettably, the overuse of antibiotics has hastened the rise of antimicrobial resistance (AMR). This is a severe public health issue and a worldwide menace, particularly in light of COVID-19. According to estimates, drug-resistant infections will cause approximately ten million deaths annually by 2050 [1]. Numerous recent reports have highlighted a surge in multidrug-resistant organisms during the COVID-19 pandemic [[2], [3], [4], [5], [6], [7], [8]]. Additionally, AMR also presents a significant issue in Guangdong, China [9]. As per the Status Report on Antimicrobial Administration and Antimicrobial Resistance in China (2022), Guangdong province has a high rate of antibiotic use (AUR) among tertiary comprehensive inpatients in 2021, as well as a high density of antibiotic use (AUD) in core data hospitals in 2021. It is crucial to address this issue, particularly in light of COVID-19, by implementing antimicrobial stewardship (AMS) measures.

The Infectious Diseases Society of America (IDSA) stated in 2012 that AMS encompasses coordinated interventions intended to enhance and assess the judicious use of antimicrobial agents. This is achieved by promoting the selection of an optimal antimicrobial drug regimen, which includes appropriate dosing, duration of therapy, and administration route [10].

Similarly, the Chinese Ministry of Health launched a long-term national AMS campaign in 2011 [11]. The campaign protocol mainly comprised setting targets for antimicrobial management, implementing educational program and prescription audit, establishing financial incentive, and the Chinese Ministry of Health requiring local health authorities to formulate interventions based on local conditions. Furthermore, the AMS policy of China was implemented in 2012, and the guidelines for clinical application of antimicrobials were updated in 2015 [12]. Many previous studies have shown that some of the measures in this AMS policy have been associated with appropriate antibiotic use, reduced prevalence of antibiotic-resistant pathogens, and improved clinical outcomes [[13], [14], [15], [16], [17]].

Generally, numerous published studies have shown that implementing AMS can considerably decrease antimicrobial consumption, costs, and adverse drug events [[18], [19], [20], [21]], including the implementation of financial incentives, pay-for-performance and penalties [[22], [23], [24], [25], [26], [27], [28]]. However, such studies have mainly been conducted in primary health care and rarely in tertiary hospitals. As a result, we initiated an AMS with pay-for-performance at a tertiary hospital to investigate its effectiveness.

This study aimed to use the interrupted time series (ITS) analysis, the strongest and quasi-experimental approach for evaluating longitudinal effects of intervention [29], to evaluate the impact of the AMS with pay-for-performance in a large teaching hospital in Guangzhou on antimicrobial consumption providing constructive policy suggestions for future AMS in China. We hypothesized that this intervention may help to optimize the use of antibiotics.

## 2 Materials and methods

### 2.1 Setting and study design

This study was carried out at an academic teaching hospital with 3956 beds located in Guangzhou, China. Antimicrobial consumption was a crucial target for the AMS, and data were extracted from the hospital information system and analyzed for this purpose. Specifically, monthly antibiotic use density (AUD) and antibiotic use rate (AUR) data were collected from each inpatient department between January 2018 and September 2022. Additionally, monthly antibiotic expenditure (AE) data for the entire hospital and total medication expenditure (ME) data for the entire hospital were collected from January 2018 to June 2022.

### 2.2 Antimicrobial stewardship

The pay-for-performance based AMS was officially implemented in April 2021 and distributed to all departments in the form of documents. Bonus payment delivered to departments for antimicrobial consumption not beyond a specified threshold and penalties imposed on departments for antimicrobial consumption beyond a specified threshold. And the supplementary materials (Table 1) provide those specified thresholds for each clinical department and outline the specific implementation details of pay-for performance. Feedback on antimicrobial consumption indicators was provided in the following two forms: (1) in the form of envelopes, this feedback was given once a month; (2) Remind departments with serious use of antibiotics to carry out rectification at the Pharmaceutical Affairs Committee (all middle-level and above cadres of the hospital participated); The Pharmaceutical Affairs Committee is held quarterly. In addition, hospitals implemented multiple soft AMS, including education and training initiatives, throughout the study period, both before and after the intervention. However, it is important to note that the implementation of these soft measures was not altered in any way by the introduction of the pay-for-performance. The objective of these initiatives is to not only guide clinicians in the rational use of antimicrobials but also to establish a basis for implementing stricter measures, such as pay-for-performance. By combining soft measures with hard measures, the goal is to foster a culture of responsible antibiotic use within the healthcare profession. These combined efforts are designed to address the issue comprehensively and promote the appropriate utilization of antibiotics.

**Table 1:** The inpatient departments analyzed.

Internal Medicine System	Surgical System	Intensive Care Unit (ICU) System
Department of Hematology, Unit 1	Neurosurgery	Medical ICU
Department of Hematology, Unit 2	Biliary and Pancreatic Surgery	Pediatric ICU
Department of Hematology, Unit 3	Department of Burn, Wound Repair& Reconstruction	Neurology ICU
Respiratory and Critical Illness	Urology	ICU, Unit 1
Rheumatic Immunology	Thoracic Surgery	ICU, Unit 2
Department of Pediatrics, Unit 2	Gastrointestinal Surgery, Unit 1	Neurosurgery ICU
Department of Cardiology, Unit 6	Gastrointestinal Surgery, Unit 2	Cardiothoracic Surgery ICU
Cardiovascular Pediatrics	Gastrointestinal Surgery, Unit 3	Emergency ICU
Dermatology	Gynecology	
Emergency Ward	Cardiac Surgery	
Special Medical and Health Management Center	Microsurgery, Trauma and Hand Surgery	
	Organ Transplant Unit	
	Oral and Maxillofacial Surgery	

### 2.3 Ethical consideration

The study did not require ethical approval because the patient's privacy was not violated in the study. And it did not include any interventions that required the use of human subjects.

### 2.4 Study outcomes

In this study, we selected three metrics to evaluate inpatient antimicrobial consumption: AUD, AUR, and the percentage of antibiotic cost (AE/ME).

In this study, the three metrics and calculation methods are as follows:

$$\text{AUD}\;\left(\text{DDDs}/100\text{PD}\right)=\frac{cumulative\;DDDs}{number\;of\;days\;of\;patients\;admitted\;during\;the\;same\;period}*100$$

$$\text{AUR}\;\left(\%\right)=\frac{number\;of\;discharged\;patients\;used\;antibiotics}{number\;of\;discharged\;patients\;during\;the\;same\;period}*100$$

$$\text{Percentage}\;\text{of}\;\text{antimicrobial}\;\text{expenditure}\;\left(\%\right)=\frac{antibiotic\;expenditure\;\left(AE\right)}{medication\;expenditure\;\left(ME\right)during\;the\;same\;period}*100$$

### 2.5 Analysis of department selection

Because of the large number of departments in the hospital, we selected representative departments for analysis based on data availability and the setting of penalty thresholds of AUD greater than 40 DDDs/100PD, which aligns with the global average AUD [30]. This is also in line with the guidelines outlined in the “Notice on Continuing to do a good job in the management of Clinical application of antibacterial Agents” (No. 8 [2020] of the State Health Office) which states that the AUD in inpatients in general tertiary hospitals should be controlled under 40 DDDs/100PD. By doing so, we aimed to assess the impact of the intervention in departments that pull up the AUD of hospital-wide, and in which departments the results of the intervention would be more pronounced. The screening results can be found in Table 1. In addition, Departments that could not be analyzed due to missing values included: Department of Hematology, Unit 2, Gastrointestinal Surgery, Unit 1 and Emergency ICU.

### 2.6 Statistical analysis

We will provide a brief description of the ITS analysis. Segmented linear regression (SLR) was used to conduct the analysis, and the formula for the model is as follows:

*Y*_*t*_ = *β*_0_+*β*_1_ × time+*β*_2_ × intervention+*β*_3_ × post-time +*β*_41_ × covid19_1+ *β*_42_ × covid19_2+ seasonality + ε

Where *Y*_t_ represents the main outcome indicator, time is a continuous variable ranging from 1 to 57 in this study, intervention is a dummy variable assigned a value of 0 before the intervention and 1 after the intervention, post-time is a variable that calculates the number of months after the intervention (with a value of 0 before the intervention and ranging from 0 to 17 after the intervention), and ε is a random variable; COVID-19 is a dummy variable indicating the pre-COVID-19 period (April 2019 to December 2019, coded 0), COVID-19 period (January 2020 to March 2020, coded 1), and post-COVID-19 (coded 2) [31]; *β*_0_ represents the baseline level of the outcome when time = 0, *β*_1_ represents the baseline trend before intervention, *β*_2_ represents the change in level following the intervention, *β*_3_ represents the change in trend following the intervention, *β*_41_ is the level change during the COVID-19 period, and *β*_42_ is the level change post-COVID-19. The final trend of the outcome after intervention is represented by the sum of *β*_1_ and *β*_3_. If necessary, we used harmonic terms specifying two sine and cosine pairs to adjust for seasonality. The Durbin Watson (DW) method was employed to detect the presence of first-order auto-correlation in the time series. In case such correlation was detected, we used the feasible generalized least square (FLGS) method to adjust for the first-order auto-correlation errors, implemented with the Cochrane-Orcutt estimation.

Statistical analysis was conducted using R 4.2.2 (Vienna, Austria), and all tests were two-sided with significance determined at a *P*-value of less than 0.05.

## 3 Results

### 3.1 Overall changes in antimicrobial consumption in hospital-wide inpatient departments due to AMS

Table 2 shows that there was a significant reduction in average AE/ME after the implementation of the intervention, decreasing from 11.02 % to 2.42 % (*P* < 0.001). However, AUD remained relatively stable (from 45.89 DDDs/100PD to 44.99 DDDs/100PD) compared to the pre-intervention period (*P* = 0.38). Similarly, no significant increase was observed in AUR after the intervention (increasing from 38.64 % to 39.17 %, *P* = 0.24).

**Table 2:** Overall changes of total hospital.

Indicators	Before intervention	After intervention	*P*-Value
AUD (DDDs/100PD)	45.89	44.99	0.38
AUR (%)	38.64	39.17	0.24
AE/ME (%)	11.02	2.42	<0.001

### 3.2 ITS analysis of total hospital

**Fig. 1:**
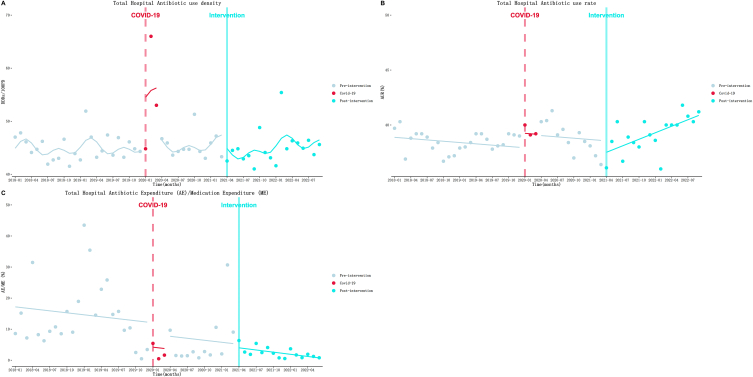
Results of the ITS analysis of total hospital antibiotic use density (A), antibiotic use rate (B) and antibiotic expenditure/medication expenditure (C).

As Table 3 and Fig. 1B showed, AUR decreased at a rate of 0.04 % (*P* = 0.37) per month before the intervention. At the beginning of the intervention, AUR decreased to a level of 1.04 % (*P* = 0.20). After that, the downward trend turned into an upward trend at a rate of 0.20 % (*P* = 0.004) per month. But, the changes of AUD and AE/ME due to intervention were not statistically significant (Table 3).

**Table 3:** Results of the ITS analysis of total hospital.

Department	β_1_ (SE)	*P*	β_2_ (SE)	*P*	β_3_ (SE)	*P*	β_1_+β_3_	Parameters of model fit	
								Dw	R^2^
Antibiotic use density (AUD)	0.02(0.07)	0.80	−1.79 (1.30)	0.17	0.14 (0.12)	0.24	0.16	2.25	0.61
Antibiotic Use Rate (AUR)	−0.04 (0.04)	0.37	−1.04 (0.81)	0.20	0.24 (0.08)	**0.004**	0.20	2.00	0.21
AE/ME	−0.21 (0.34)	0.53	−1.15 (6.35)	0.86	−0.02 (0.74)	0.98	−0.23	2.08	0.20

### 3.3 ITS analysis of Internal Medicine system AUD

**Fig. 2:**
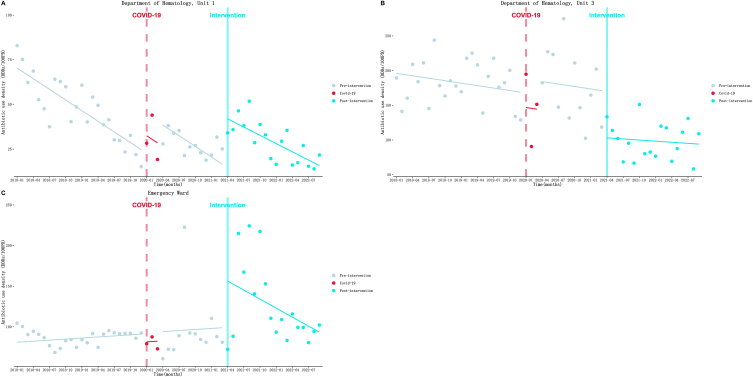
Results of the ITS analysis of Internal Medicine system antibiotic use density. A: Department of Hematology, Unit 1; B: Department of Hematology, Unit 3;C: Emergency Ward.

Table 4 and Fig. 2 demonstrate the results of the ITS analysis, which are as follows: (1) Department of Hematology, Unit 1 (Fig. 2A): Before implementing the intervention, AUD decreased at a rate of 1.99 DDDs/100PD (*P* < 0.001) per month. However, an immediate increase of 27.35 DDDs/100PD (*P* < 0.001) was observed at the beginning of the intervention. After the intervention, the decline trend slowed down, with a rate of 1.54 DDDs/100PD (*P* = 0.33) per month. (2) Department of Hematology, Unit 3 (Fig. 2B): During the pre-intervention period, AUD decreased at a rate of 1.18 DDDs/100PD (*P* = 0.25) per month. Then, an immediate and substantial decline of 66.93 DDDs/100PD (*P* = 0.002) was observed in the first month of the intervention. After that, the declining trend appeared to change minimally, at a rate of 0.53 DDDs/100PD (*P* = 0.74) per month. (3) Emergency Ward (Fig. 2C): Before the intervention, AUD increased at a rate of 0.43 DDDs/100PD (*P* = 0.71) per month. However, at the beginning of the intervention, AUD continued to increase, reaching a level of 57.00 DDDs/100PD (*P* = 0.01) immediately. After that, the increasing trend turned into a decreasing trend, declining at a rate of 3.70 DDDs/100PD (*P* = 0.06) per month. Additionally, the changes in other departments of the Internal Medicine system due to the intervention were not statistically significant. A detailed summary of these results can be found in the supplementary materials (Table S2 and Fig. S1).

**Table 4:** Results of the ITS analysis of AUD.

Department	β_1_ (SE)	*P*	β_2_ (SE)	*P*	β_3_ (SE)	*P*	β_1_+β_3_	Parameters of model fit	
								Dw	R^2^
**Internal Medicine System**									
Department of Hematology, Unit 1	−1.99 (0.23)	**<0.001**	27.35 (4.79)	**<0.001**	0.45 (0.45)	0.33	−1.54	1.86	0.76
Department of Hematology, Unit 3	−1.18 (1.00)	0.25	−66.93 (20.47)	**0.002**	0.65 (1.93)	0.74	−0.53	2.55	0.56
Emergency Ward	0.43 (1.16)	0.71	57.00 (21.70)	**0.01**	−4.13 (2.11)	0.06	−3.70	2.03	0.23
**Surgical System**									
Urology	0.40 (0.16)	**0.02**	−32.80 (3.36)	**<0.001**	−0.19 (0.32)	0.55	0.21	2.08	0.79
Gastrointestinal Surgery, Unit 3	0.29 (0.26)	0.27	−11.44 (5.25)	**0.03**	1.39 (0.49)	**0.007**	1.68	1.70	0.36
Cardiac Surgery	0.48 (0.27)	0.08	−14.30 (5.53)	**0.01**	−0.08 (0.52)	0.87	0.40	1.67	0.22
Organ Transplant Unit	1.42 (0.37)	**<0.001**	−10.53 (7.53)	0.17	−1.64 (0.71)	**0.02**	−0.22	2.07	0.36
**ICU System**									
Pediatric ICU	−3.41 (0.90)	**<0.001**	14.75 (18.24)	0.42	4.23 (1.72)	**0.02**	0.82	2.22	0.33
Neurology ICU	0.13 (1.12)	0.91	45.22 (22.84)	**0.05**	−2.55 (2.15)	0.24	−2.42	2.57	0.11
ICU, Unit 2	1.80 (1.67)	0.29	−81.91 (34.07)	**0.02**	1.29 (3.20)	0.69	3.09	2.54	0.18
Cardiothoracic Surgery ICU	2.51 (1.02)	**0.02**	−41.52 (20.83)	**0.05**	0.67 (1.96)	0.73	3.18	2.17	0.22

### 3.4 ITS analysis of surgical system AUD

**Fig. 3:**
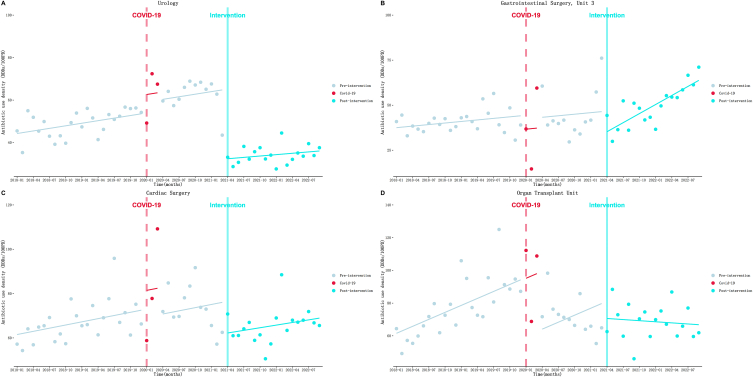
Results of the ITS analysis of Surgical system antibiotic use density. A: Urology; B: Gastrointestinal Surgery, Unit 3; C: Cardiac Surgery; D: Organ Transplant Unit.

In Table 4 and Fig. 3, the outcomes are presented. (1) Urology (Fig. 3A): During the pre-intervention period, AUD increased at a rate of 0.40 DDDs/100PD (*P* = 0.02) per month. However, after introducing the intervention, there was an immediate and substantial decrease of 32.80 DDDs/100PD (*P* < 0.001). After the intervention, although not statistically significant, the increasing trend appeared to decrease slightly at a rate of 0.21 DDDs/100PD (*P* = 0.55) per month. (2) Gastrointestinal Surgery, Unit 3 (Fig. 3B): Before the intervention, no significant increase in AUD was observed at a rate of 0.29 DDDs/100PD (*P* = 0.27) per month. However, in the first month of the intervention, AUD immediately decreased by 11.44 DDDs/100PD (*P* = 0.03). After the intervention, the increasing trend accelerated to a rate of 1.68 DDDs/100PD (*P* = 0.007) per month. (3) Cardiac Surgery (Fig. 3C): AUD increased at a rate of 0.48 DDDs/100PD (*P* = 0.08) per month before the intervention. As expected, there was an immediate decrease in AUD by 14.30 DDDs/100PD (*P* = 0.01) in the first month of the intervention. After the intervention, the increasing trend appears to have slowed, increasing by 0.40 DDDs/100PD (*P* = 0.87) per month. (4) Organ Transplant Unit (Fig. 3D): Before implementing the intervention, AUD increased at a rate of 1.42 DDDs/100PD (*P* < 0.001) per month. At the onset of the intervention, AUD immediately declined to a level of 10.53 DDDs/100PD (*P* = 0.17). Following this, the increasing trend turned into a decreasing trend at a rate of 1.64 DDDs/100PD (*P* = 0.02) per month. Additionally, the supplementary materials (Table S2 and Fig. S2) contain the results of other departments within the Surgery system.

### 3.5 ITS analysis of ICU system AUD

**Fig. 4:**
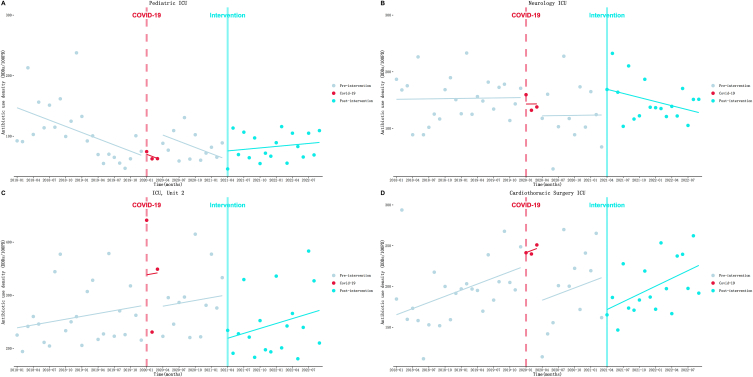
Results of the ITS analysis of ICU System antibiotic use density. A: Pediatric ICU; B: Neurology ICU; C: ICU, Unit 2; D: Cardiothoracic Surgery ICU.

As displayed in Table 4 and Fig. 4. (1) Pediatric ICU (Fig. 4A): AUD decreased at a rate of 3.41 DDDs/100PD (*P* < 0.001) per month before the intervention was implemented. Then an immediate rise of 14.75 DDDs/100PD (*P* = 0.42) was observed at the beginning month of the intervention. After the intervention, the downward trend turned into an upward trend at a rate of 0.82 DDDs/100PD (*P* = 0.02). (2) Neurology ICU (Fig. 4B): AUD increased at a rate of 0.13 DDDs/100PD (*P* = 0.91) per month before the intervention was implemented. Then an immediate rise of 45.22 DDDs/100PD (*P* = 0.05) was observed at the beginning month of the intervention. After the intervention, the upward trend appeared to turn into a downward trend at a rate of 2.42 DDDs/100PD (*P* = 0.24) per month. (3) ICU, Unit 2 (Fig. 4C): An increasing trend at a rate of 1.80 DDDs/100PD (*P* = 0.29) per month was observed in the pre-intervention period. Then AUD instantly decreased by 81.91 DDDs/100PD (*P* = 0.02) in the first month of intervention. After that, the increasing trend seemed to accelerate slightly to a rate of 3.09 DDDs/100PD (*P* = 0.69) per month. (4) Cardiothoracic Surgery ICU (Fig. 4D): An increasing trend at a rate of 2.51 DDDs/100PD (*P* = 0.02) per month was observed in the pre-intervention period. However, AUD instantly decreased by 41.52 DDDs/100PD (*P* = 0.05) in the first month of intervention. After that, the increasing trend seemed to accelerate slightly to a rate of 3.18 DDDs/100PD (*P* = 0.73) per month. Furthermore, the results of other departments of ICU system can be found in the supplementary materials (Table S2 and Fig. S3).

### 3.6 ITS analysis of Internal Medicine system AUR

**Fig. 5:**
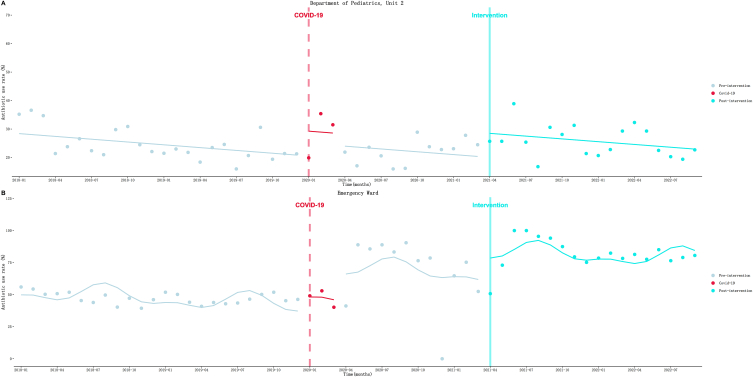
Results of the ITS analysis of Internal Medicine system antibiotic use rate. A: Department of Pediatrics, Unit 2; B: Emergency Ward.

As shown in Table 5 and Fig. 5. (1) Department of Pediatrics, Unit 2 (Fig. 5A): AUR decreased at a rate of 0.33 % (*P* = 0.03) per month before the intervention. Then an immediate increase of 8.44 % (*P* = 0.006) was observed in the first month of the intervention. After that, the decreasing trend seemed to accelerate slightly to a rate of 0.327 % (*P* = 0.99) per month. (2) Emergency Ward (Fig. 5B): a gradually decrease in AUR at a rate of 0.50 % *(P* = 0.22) per month was observed before the intervention was implemented. However, at the beginning of the intervention, AUR increased to a level of 18.42 % (*P* = 0.02) immediately. After that, the downward trend seemed to accelerate slightly to a rate of 0.36 % (*P* = 0.85) per month. ITS analysis results of other departments of Internal Medicine system can be found in the supplementary materials (Table S3 and Fig. S4).

**Table 5:** Results of the ITS analysis of AUR.

Department	β_1_ (SE)	*P*	β_2_ (SE)	*P*	β_3_ (SE)	*P*	β_1_+β_3_	Parameters of model fit	
								Dw	R^2^
**Internal Medicine System**									
Department of Pediatrics, Unit 2	−0.33 (0.14)	**0.03**	8.44 (2.91)	**0.006**	0.003 (0.27)	0.99	−0.327	1.71	0.20
Emergency Ward	−0.50 (0.40)	0.22	18.42 (7.87)	**0.02**	0.14 (0.74)	0.85	−0.36	1.83	0.64
**Surgical System**									
Urology	0.13 (0.13)	0.33	−6.56 (2.62)	**0.02**	0.04 (0.25)	0.86	0.17	2.58	0.21
Gastrointestinal Surgery, Unit 3	−0.01 (0.16)	0.95	−8.50 (3.23)	**0.01**	0.84 (0.30)	**0.008**	0.83	1.93	0.47
Microsurgery, Trauma and Hand Surgery	0.33 (0.20)	0.10	8.01 (4.01)	**0.05**	0.07 (0.38)	0.85	0.40	2.01	0.56
**ICU System**									
Pediatric ICU	0.06 (0.52)	0.92	−8.73 (8.69)	0.32	−1.88 (0.95)	**0.05**	−1.82	1.73	0.32

### 3.7 ITS analysis of surgical system AUR

**Fig. 6:**
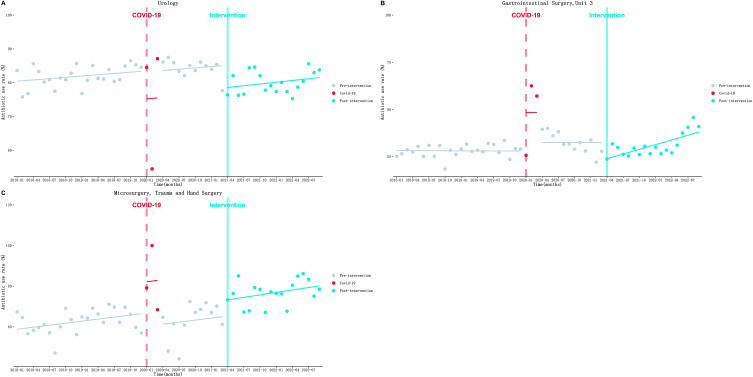
Results of the ITS analysis of Surgical system antibiotic use rate. A: Urology; B: Gastrointestinal Surgery, Unit 3; C: Microsurgery.

Table 5 and Fig. 6 illustrates the results. (1) Urology (Fig. 6A): a increasing trend at a rate of 0.13 % (*P* = 0.33) per month of AUR was observed during the pre-intervention. Then an immediate decrease to a level of 6.56 % (*P* = 0.02) was observed at the beginning of the intervention. After that, the upward trend seemed to accelerate slightly to a rate of 0.17 % (*P* = 0.86) per month. (2) Gastrointestinal Surgery, Unit 3 (Fig. 6B): AUR gradually decreased at a rate of 0.01 % (*P* = 0.95) per month during the pre-intervention period. At the beginning of the intervention, AUR dropped greatly by 8.50 % immediately (*P* = 0.01). But after that, the decreasing trend significantly turned to upward trend at a rate of 0.83 % (*P* = 0.008) per month. (3) Microsurgery (Fig. 6C): AUR gradually increased at a rate of 0.33 % (*P* = 0.10) per month before the intervention. Then an immediate increase to a level of 8.01 % (*P* = 0.05) was observed at the beginning of the intervention. After that, the upward trend seemed to accelerate slightly to a rate of 0.40 % (*P* = 0.85) per month. Other surgery system departments’ ITS results can be found in the supplementary materials (Table S3 and Fig. S5).

### 3.8 ITS analysis of ICU system AUR

**Fig. 7:**
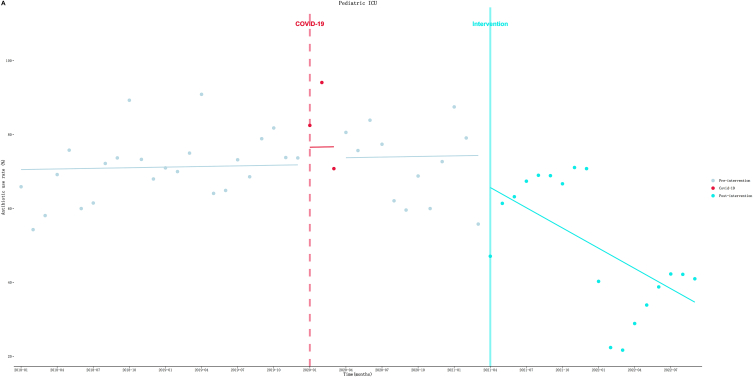
Results of the ITS analysis of ICU system antibiotic use rate.

As shown in Table 5 and Fig. 7. (1) Pediatric ICU: AUR seemed to gradually increase at a rate of 0.06 % (*P* = 0.92) per month before the intervention. Then an immediate drop of AUR to a level of 8.73 % (*P* = 0.32) was observed in the first month of the intervention. After that, the upward trend turned to downward trend at a rate of 1.83 % (*P* = 0.05) per month. Other ICU system departments’ ITS results can be found in the supplementary materials (Table S3 and Fig. S6).

## 4 Discussion

In general, our findings indicate a significant short-term effect of pay-for-performance based AMS in reducing antimicrobial consumption across many clinical departments within a large teaching hospital in Guangzhou, China. However, we only observe a lasting impact of this intervention on reducing antimicrobial consumption in a few departments. It is important to highlight that our study did not identify a significant impact of the intervention on reducing the AUD and AE/ME at the hospital. And surprisingly, the intervention seemed to contribute to an upward trend in AUR throughout the hospital.

We noted a significant reduction in the percentage of hospital-wide antibiotic cost (AE/ME) in pre-post-comparisons. Interestingly, however, this significant reduction was not present in the ITS analysis results. Perhaps we can interpret this phenomenon based on the intervention itself. The intervention solely established thresholds for AUD and AUR without making any assertions regarding the cost of antimicrobials. Different from our study, Qian et al. reported that AMS which included setting a specific target for antimicrobial use was associated with the long-term trend of AE/ME and slowed the pre-intervention decline trend in AE/ME in inpatients [32]. We believe that this difference may be associated with the relatively single interventions in our study. In the study conducted by Qian et al. they implemented several interventions, which encompassed not only rewards and punishments but also the establishment of an antibiotic control system. This system was then integrated into the existing hospital information system.

Although our study found that the intervention had no significant effect on the overall AUD in the hospital, and was even associated with an upward trend in the overall AUR in the hospital, the analysis of specific key departments showed that the intervention led to a decline in antimicrobial consumption in some departments. Given the overuse of antibiotics and the resistance associated with overuse in China, these results are encouraging.

For certain departments, the intervention initially resulted in a notable and immediate decrease in antimicrobial consumption. In reality, research in behavioral economics and public health has demonstrated that financial incentives and penalties are generally considered robust tools for altering provider behaviors [33]. However, the long-term impact of intervention did not show significant results or the decline in antimicrobial consumption gradually slowed down during the post-intervention period. This was observed in departments such as Department of Hematology, Unit 3, Gastrointestinal Surgery, Unit 3, and so on. To our knowledge, this may be mainly attributed to reducing power at different stages. Initially, the high antimicrobial consumption at baseline provided ample room for reduction. When antimicrobial consumption reaches low levels, there is little room for further reductions. Similar findings were also observed in other AMS reports. For example, a study found no significant change in antimicrobial consumption in a Tertiary Women's and Children's Hospital following AMS because of the low base levels of antimicrobial consumption in the institution, even though the primary AMS strategy employed in the study was a prospective audit and feedback approach [34].

In contrast to the significant impact of the intervention on a number of departments in the short term, the majority of departments in the hospital were relatively unaffected (statistically insignificant) by the intervention. We believe that one possible reason is that department leaders value performance in other areas, such as surgery. And they don't care about fines for substandard antimicrobial indicators.

Overall, our findings are generally consistent with previous studies that have demonstrated this intervention's effectiveness in reducing antimicrobial consumption. Gong, S et al. have reported that a significant decline in antibiotic use and corresponding expenditure in both ambulatory and inpatient clinical settings after the inclusion of bonus to an AMS based on prior authorization alone [35]. Borek et al. reported that bonus can optimize antibiotic prescribing in primary care general practices throughout England [26]. Balinskaite et al. demonstrated that the introduction of bonus for local healthcare commissioners was associated with a significant reduction in both total and broad-spectrum antibiotic prescribing in primary care throughout England [27]. Martens, J. D et al. also reported that behavior independent bonus can be a help in changing prescription behavior of general practitioners, and effects are small-scale and temporary [28].

The preliminary results of our study are very promising and motivate us to continue our efforts to reduce AUR and AUD. In addition, given the short-term impact of our findings on antimicrobial consumption, we recommend implementing such hard measures in departments where there is considerable room for improvement in antimicrobial use, such as those with high baseline antimicrobial use. In such cases, such hard measures are more likely to help reduce antimicrobial consumption.

Several limitations associated with this study must be acknowledged. Firstly, it is a retrospective study conducted at a single center without a control group. Therefore, our findings may not be directly generalizable to other settings. Although we applied ITS analysis to minimize internal validity threats, we cannot ensure that the pay-for-performance based AMS were solely responsible for the changes reported in our findings. Therefore, A prospective multi-center study with a control group is necessary to generalize the utility of the intervention in the future. Secondly, we only examined changes in AUD, AUR, and economic indicators. However, these indicators do not directly reflect the appropriateness of antibiotic prescribing. Finally, due to the lack of case data for individual patients, we were unable to analyze clinical outcomes after re-intervention or control for other factors that might affect changes in antibiotic use. Despite these limitations, this study can still serve as a reference for further research aimed at addressing these limitations.

## 5 Conclusion

The pay-for-performance based AMS proved effective in reducing antibiotic consumption in certain departments of a large teaching hospital in Guangzhou, particularly in the short-term period. Future studies will be necessary to verify its effectiveness, identify areas for improvement, and establish evidence on the causal mechanisms that incentivize doctors' prescribing patterns for antibiotics. Furthermore, our study serves as a useful reference for other hospitals looking to implement similar antimicrobial stewardship programs, including in which departments and the specific details of implementation. This strategy is simple, economical, and feasible, and its replication in other healthcare settings could prove beneficial in addressing the issue of antibiotic resistance.

## Acknowledgments and funding statement

We would like to thank Qiuyi He, Jiajia Yan, Yanzhe Xia, Dr. Jiawei Zeng, Dr.Liyan Zhao, Dr. Pan Chen for their professional work on antimicrobial stewardship programme.

The study was funded by the 10.13039/501100001809National Natural Science Foundation of China [grant number: 72074234, 72374228]; 10.13039/501100021171Guangdong Basic and Applied Basic Research Foundation [grant number: 2022A1515011338, 2023A1515010163], Guangzhou Basic and Applied Basic Research Program [grant number: 202201011208].

## Data availability statement

The materials and datasets analyzed during the current study are available from the corresponding author on reasonable request.

## CRediT authorship contribution statement

**Haohai Xia:** Writing – original draft, Formal analysis, Conceptualization. **Jia Li:** Writing – review & editing, Data curation, Conceptualization. **Xinyi Yang:** Writing – review & editing, Formal analysis. **Yingchao Zeng:** Formal analysis. **Lin Shi:** Formal analysis. **Weibin Li:** Formal analysis. **Xu Liu:** Writing – review & editing. **Shifang Yang:** Writing – review & editing. **Manzhi Zhao:** Writing – review & editing. **Jie Chen:** Writing – review & editing, Data curation. **Lianping Yang:** Writing – review & editing, Project administration, Funding acquisition, Conceptualization.

## Declaration of competing interest

The authors declare that they have no known competing financial interests or personal relationships that could have appeared to influence the work reported in this paper.
