# Supplementary material for: Effects of pay-for-performance based antimicrobial stewardship on antimicrobial consumption and expenditure: An interrupted time series analysis
Source: Heliyon. 2024 Jun 8;10(12):e32750. doi: 10.1016/j.heliyon.2024.e32750 (PMC11226823; doi:10.1016/j.heliyon.2024.e32750)
Supplement: Multimedia component 1 [file mmc1.docx]

**Supplementary materials**

According to the consumption of antibiotics in various department in the past three years, the rationality evaluation of antimicrobial prescriptions by clinical pharmacists in various departments, and the performance appraisal requirements of national tertiary public hospitals-"Notice on Strengthening the Management of Clinical Application of Antibacterial Drugs" (National Health Commission, Medical Administration [2015] No. 42) ", the pharmaceutical affairs committee of the hospital has set thresholds for different departments after many discussions. The indicators for the rational clinical use of antibiotics in hospitalized departments are stipulated as follows:

| **Table S1 Indicators requirements for rational clinical use of antibiotics in inpatient departments** | | | | |
| --- | --- | --- | --- | --- |
| **Department** | **AUR (%)** | | **AUD (DDDs/100PD)** | |
|  | **Lower limit** | **Upper limit** | **Lower limit** | **Upper limit** |
| **Internal Medicine system** |  |  |  |  |
| Department of Hematology, Unit 1 | 30 | 35 | 60 | 70 |
| Department of Hematology, Unit 2 | 30 | 35 | 60 | 65 |
| Department of Hematology, Unit 3 | 60 | 65 | 150 | 200 |
| Respiratory and Critical Illness | 45 | 50 | 75 | 90 |
| Rheumatic Immunology | 35 | 40 | 40 | 45 |
| Department of Pediatrics, Unit 2 | 30 | 35 | 80 | 95 |
| Department of Cardiology,  Unit 6 | 30 | 35 | 40 | 50 |
| Cardiovascular Pediatrics | 55 | 60 | 45 | 50 |
| Dermatology | 40 | 45 | 50 | 60 |
| Emergency Ward | 55 | 65 | 65 | 75 |
| Special Medical and Health Management Center | 30 | 35 | 50 | 60 |
| **Surgical system** |  |  |  |  |
| Neurosurgery | 70 | 75 | 50 | 55 |
| Biliary and Pancreatic Surgery | 60 | 75 | 35 | 45 |
| Department of Burn, Wound Repair& Reconstruction | 85 | 90 | 40 | 50 |
| Urology | 75 | 85 | 45 | 50 |
| Thoracic Surgery | 35 | 45 | 45 | 50 |
| Gastrointestinal Surgery, Unit 1 | 40 | 45 | 45 | 55 |
| Gastrointestinal Surgery, Unit 2 | 40 | 45 | 45 | 55 |
| Gastrointestinal Surgery, Unit 3 | 40 | 45 | 45 | 55 |
| Gynecology | 55 | 65 | 40 | 50 |
| Cardiac Surgery | 60 | 80 | 60 | 70 |
| Microsurgery, Trauma and Hand Surgery | 45 | 55 | 35 | 45 |
| Organ Transplant Unit | 50 | 55 | 60 | 80 |
| Oral and Maxillofacial Surgery | 65 | 70 | 35 | 45 |
| **Intensive Care Unit (ICU) system** |  |  |  |  |
| Medical ICU | 80 | 90 | 180 | 260 |
| Pediatric ICU | 60 | 70 | 100 | 125 |
| Neurology ICU | 65 | 70 | 100 | 135 |
| ICU, Unit 1 | 75 | 85 | 150 | 225 |
| ICU, Unit 2 | 85 | 95 | 200 | 270 |
| Neurosurgery ICU | 85 | 90 | 125 | 175 |
| Cardiothoracic Surgery ICU | 85 | 95 | 150 | 200 |
| Emergency ICU | 85 | 95 | 150 | 220 |

The specific rewards and penalty measures are as follows:

(Ⅰ) Departments whose all indexes are below the lower limit (including the lower limit) will be given a one-time reward of 15,000 yuan.

(Ⅱ) If overuse of antibiotics is found in daily inspection, the corresponding department reward will be cancelled.

(Ⅲ) Departments with one or more indicators higher than the upper limit will be given different degrees of fine according to the number of items that fail to meet the standard. The fine was derived from the salary of all clinicians with prescription rights in the department:

1. If one item fails to meet the standard, a one-time deduction of 2,000 yuan will be made.

2. If two items fail to meet the standard, a one-time deduction of 4,000 yuan will be made.

3. If three items fail to meet the standard, a one-time deduction of 6,000 yuan will be made.

| Table S2 Results of the ITS analysis of AUD | | | | | | | | | |
| --- | --- | --- | --- | --- | --- | --- | --- | --- | --- |
| Department | **β_1_**  **(SE**） | ***P*** | **β_2_**  **(SE**） | ***P*** | **β_3_**  **(SE**） | ***P*** | **β_1_+β_3_** | **Parameters of model fit** | |
|  | | | | | | | | Dw | R^2^ |
| Internal Medicine system |  |  |  |  |  |  |  |  |  |
| Respiratory and Critical Illness | 0.76  (0.27) | **0.007** | -2.00  (5.30) | 0.71 | -0.74  (0.50) | 0.15 | 0.02 | 2.13 | 0.50 |
| Rheumatic Immunology | 0.21  (0.33) | 0.53 | -9.15  (12.98) | 0.48 | 0.02  (1.10) | 0.99 | 0.23 | 2.49 | 0.16 |
| Department of Pediatrics, Unit 2 | 0.76  (1.35) | 0.58 | 15.49  (18.55) | 0.41 | -1.07  (2.53) | 0.68 | -0.31 | 2.12 | 0.05 |
| Department of Cardiology,  Unit 6 | -0.05  (0.49) | 0.91 | -4.66  (8.80) | 0.60 | 1.23  (0.85) | 0.16 | 1.18 | 1.79 | 0.27 |
| Dermatology | 0.63  (0.63) | 0.33 | -12.76  (12.92) | 0.33 | 0.36  (1.22) | 0.77 | 0.99 | 2.00 | 0.04 |
| Special Medical and Health Management Center | -0.60  (0.56) | 0.29 | -2.36  (11.38) | 0.84 | 1.01  (1.08) | 0.36 | 0.41 | 2.00 | 0.08 |
| Surgical system |  |  |  |  |  |  |  |  |  |
| Biliary and Pancreatic Surgery | 0.30  (0.17) | 0.08 | 4.83  (3.37) | 0.16 | -0.52  (0.32) | 0.11 | -0.22 | 1.91 | 0.24 |
| Neurosurgery | 0.14  (0.14) | 0.32 | -2.68  (2.87) | 0.36 | -0.03  (0.27) | 0.92 | 0.11 | 2.17 | 0.50 |
| Department of Burn, Wound Repair& Reconstruction | -0.28  (0.37) | 0.45 | -2.83  (7.54) | 0.71 | 0.58  (0.71) | 0.42 | 0.30 | 2.29 | 0.06 |
| Thoracic Surgery | 0.54  (0.23) | **0.02** | -3.96  (4.59) | 0.39 | 0.12  (0.43) | 0.78 | 0.66 | 1.95 | 0.38 |
| Gastrointestinal Surgery, Unit 2 | -0.64  (0.31) | **0.04** | 8.55  (6.30) | 0.18 | -0.31  (0.59) | 0.60 | -0.95 | 2.12 | 0.29 |
| Gynecology | -0.12  (0.15) | 0.45 | -4.97  (3.02) | 0.11 | 0.45  (0.29) | 0.12 | 0.33 | 2.42 | 0.29 |
| Microsurgery, Trauma and Hand Surgery | 0.15  (0.47) | 0.74 | 5.70  (9.47) | 0.55 | -0.56  (0.89) | 0.53 | -0.41 | 1.69 | 0.22 |
| Oral and Maxillofacial Surgery | 0.47  (0.28) | 0.10 | 6.77  (5.68) | 0.24 | -0.40  (0.53) | 0.45 | 0.07 | 2.17 | 0.75 |
| ICU system |  |  |  |  |  |  |  |  |  |
| Medical ICU | 2.70  (1.55) | 0.09 | 4.48  (31.65) | 0.89 | -1.29  (2.98) | 0.67 | 1.41 | 2.47 | 0.12 |
| ICU, Unit 1 | -0.13  (1.49) | 0.93 | -4.01  (29.56) | 0.89 | -0.11  (2.79) | 0.97 | -0.24 | 2.58 | 0.17 |
| Neurosurgery ICU | 0.45  (1.30) | 0.73 | -17.41  (26.44) | 0.51 | -1.69  (2.49) | 0.50 | -1.24 | 2.05 | 0.17 |


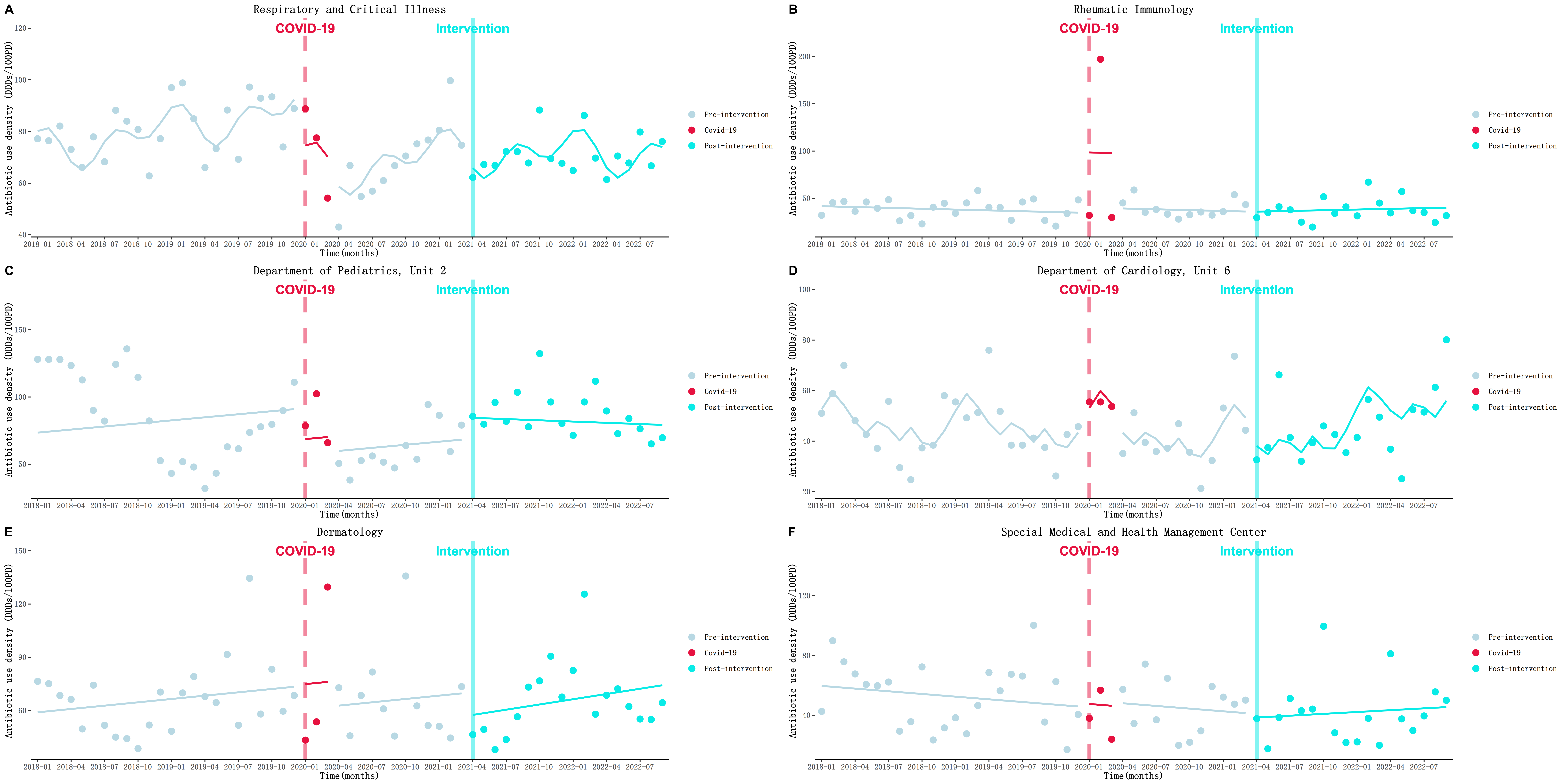


**Figure S1. Results of the ITS analysis of Internal Medicine system AUD**


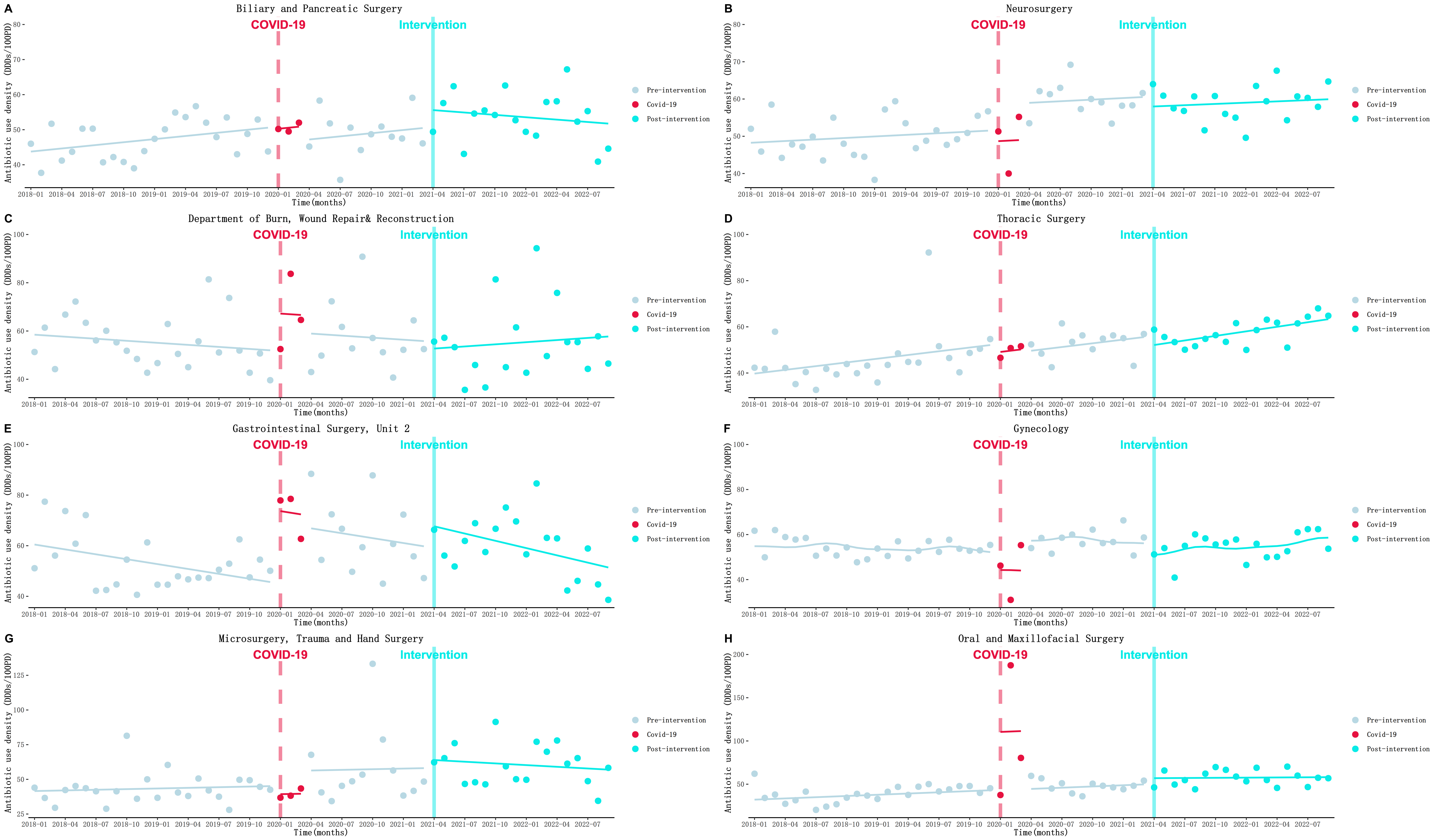


**Figure S2. Results of the ITS analysis of Surgical system AUD**


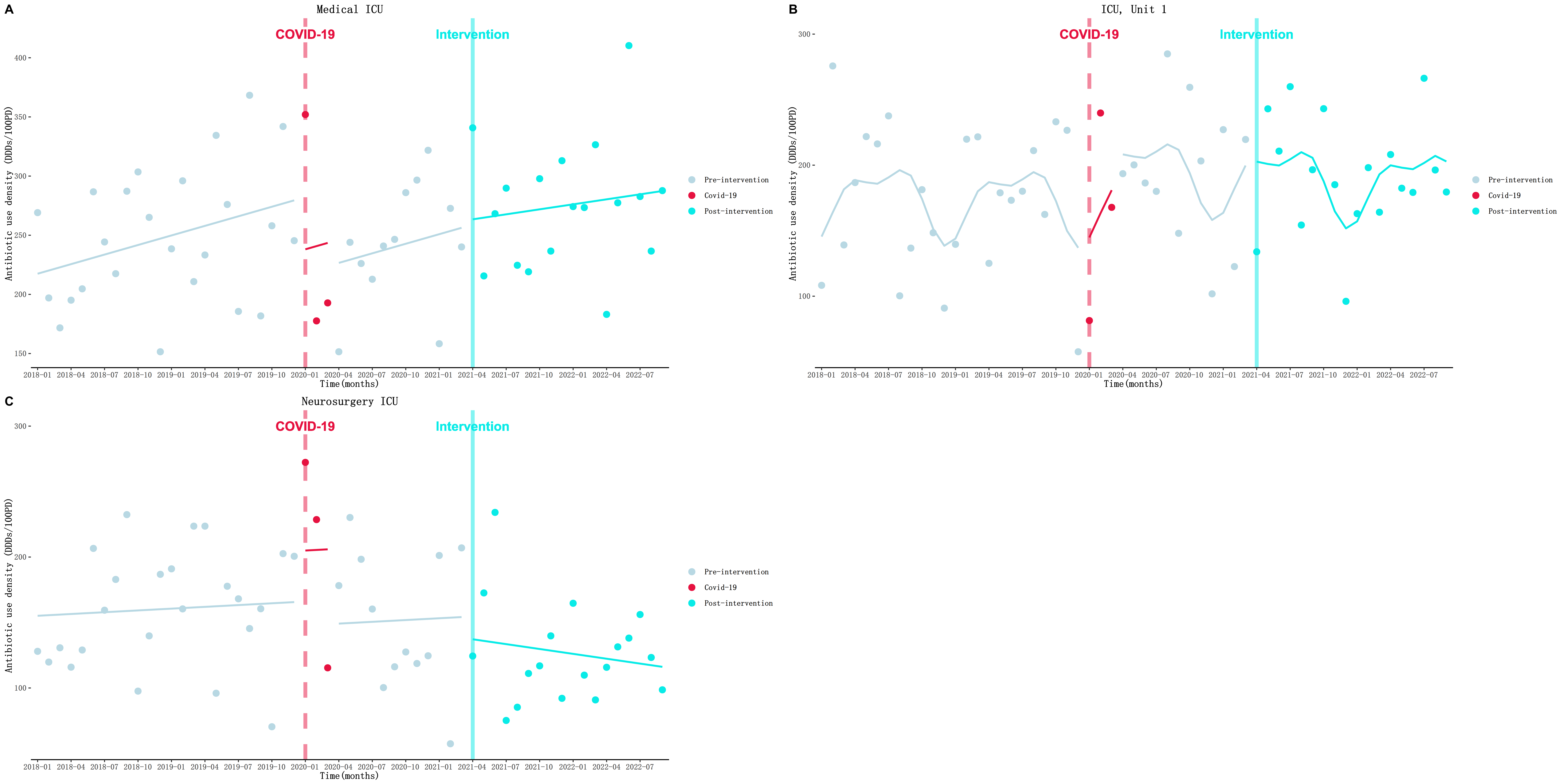


**Figure S3. Results of the ITS analysis of ICU System AUD**

| Table S3 Results of the ITS analysis of AUR | | | | | | | | | |
| --- | --- | --- | --- | --- | --- | --- | --- | --- | --- |
| Department | **β_1_**  **(SE)** | ***P*** | **β_2_**  **(SE)** | ***P*** | **β_3_**  **(SE)** | ***P*** | **β_1_+β_3_** | **Parameters of model fit** | |
|  | | | | | | | | Dw | R^2^ |
| Internal Medicine system |  |  |  |  |  |  |  |  |  |
| Department of Hematology, Unit 1 | -0.25  (0.09) | **0.006** | 2.25  (1.78) | 0.21 | -0.27  (0.17) | 0.11 | -0.52 | 1.77 | 0.77 |
| Department of Hematology, Unit 3 | 0.02  (0.24) | 0.93 | -1.64  (4.97) | 0.74 | 0.32  (0.47) | 0.50 | 0.34 | 2.27 | 0.07 |
| Rheumatic Immunology | -0.07  (0.24) | 0.78 | -3.31  (4.38) | 0.45 | 0.14  (0.43) | 0.74 | 0.07 | 1.77 | 0.08 |
| Respiratory and Critical Illness | 0.004  (0.12) | 0.97 | -3.15  (2.38) | 0.19 | 0.40  (0.22) | 0.08 | 0.404 | 1.68 | 0.72 |
| Department of Cardiology,  Unit 6 | -0.16  (0.17) | 0.35 | 5.18  (3.40) | 0.13 | 0.24  (0.32) | 0.45 | 0.08 | 2.26 | 0.18 |
| Dermatology | 0.05  (0.28) | 0.85 | -4.33  (5.22) | 0.41 | 0.80  (0.52) | 0.13 | 0.85 | 2.06 | 0.25 |
| Special Medical and Health Management Center | 0.03  (0.19) | 0.88 | -2.87  (3.72) | 0.44 | 0.07  (0.35) | 0.84 | 0.10 | 2.31 | 0.54 |
| Surgery system |  |  |  |  |  |  |  |  |  |
| Neurosurgery | -0.08  (0.13) | 0.53 | 3.84  (2.64) | 0.15 | 0.17  (0.25) | 0.49 | 0.09 | 1.94 | 0.55 |
| Biliary and Pancreatic Surgery | -0.38  (0.15) | **0.01** | 0.95  (3.05) | 0.76 | 0.35  (0.29) | 0.23 | -0.03 | 1.99 | 0.30 |
| Department of Burn, Wound Repair& Reconstruction | -0.58  (0.17) | **0.001** | -3.59  (3.50) | 0.31 | 0.63  (0.33) | 0.06 | 0.05 | 1.99 | 0.62 |
| Thoracic Surgery | 0.25  (0.16) | 0.13 | 5.84  (3.34) | 0.09 | -0.04  (0.31) | 0.90 | 0.21 | 2.11 | 0.60 |
| Gastrointestinal Surgery, Unit 2 | 0.06  (0.17) | 0.73 | -1.68  (3.54) | 0.64 | -0.003  (0.33) | 0.99 | 0.057 | 2.23 | 0.07 |
| Gynecology | 0.15  (0.15) | 0.33 | -4.53  (3.03) | 0.14 | -0.18  (0.29) | 0.53 | -0.03 | 2.58 | 0.48 |
| Cardiac Surgery | -0.01  (0.15) | 0.94 | -1.68  (3.03) | 0.58 | 0.22  (0.28) | 0.45 | 0.21 | 2.03 | 0.11 |
| Organ Transplant Unit | -0.06  (0.18) | 0.72 | 3.53  (3.57) | 0.33 | -0.07  (0.34) | 0.84 | -0.13 | 2.74 | 0.74 |
| Oral and Maxillofacial Surgery | 0.51  (0.22) | **0.03** | -6.76  (4.53) | 0.14 | -0.04  (0.43) | 0.93 | 0.47 | 1.73 | 0.23 |
| ICU system |  |  |  |  |  |  |  |  |  |
| Medical ICU | 0.26  (0.15) | 0.09 | -0.22  (2.81) | 0.94 | 0.03  (0.27) | 0.91 | 0.29 | 1.94 | 0.18 |
| ICU, Unit 1 | 0.15  (0.09) | 0.11 | 2.84  (1.87) | 0.13 | -0.31  (0.18) | 0.08 | -0.16 | 1.80 | 0.79 |
| Neurology ICU | -0.23  (0.27) | 0.41 | 7.18  (5.55) | 0.20 | 0.13  (0.52) | 0.81 | -0.10 | 1.73 | 0.11 |
| ICU, Unit 2 | 0.11  (0.07) | 0.14 | 1.00  (1.48) | 0.51 | -0.27  (0.14) | 0.06 | -0.16 | 2.00 | 0.08 |
| Neurosurgery ICU | 0.18  (0.10) | 0.07 | -0.88  (2.03) | 0.67 | -0.24  (0.19) | 0.22 | -0.06 | 1.87 | 0.27 |
| Cardiothoracic Surgery ICU | 0.22  (0.07) | **0.002** | -1.91  (1.43) | 0.19 | -0.22  (0.13) | 0.11 | 0.00 | 2.21 | 0.32 |


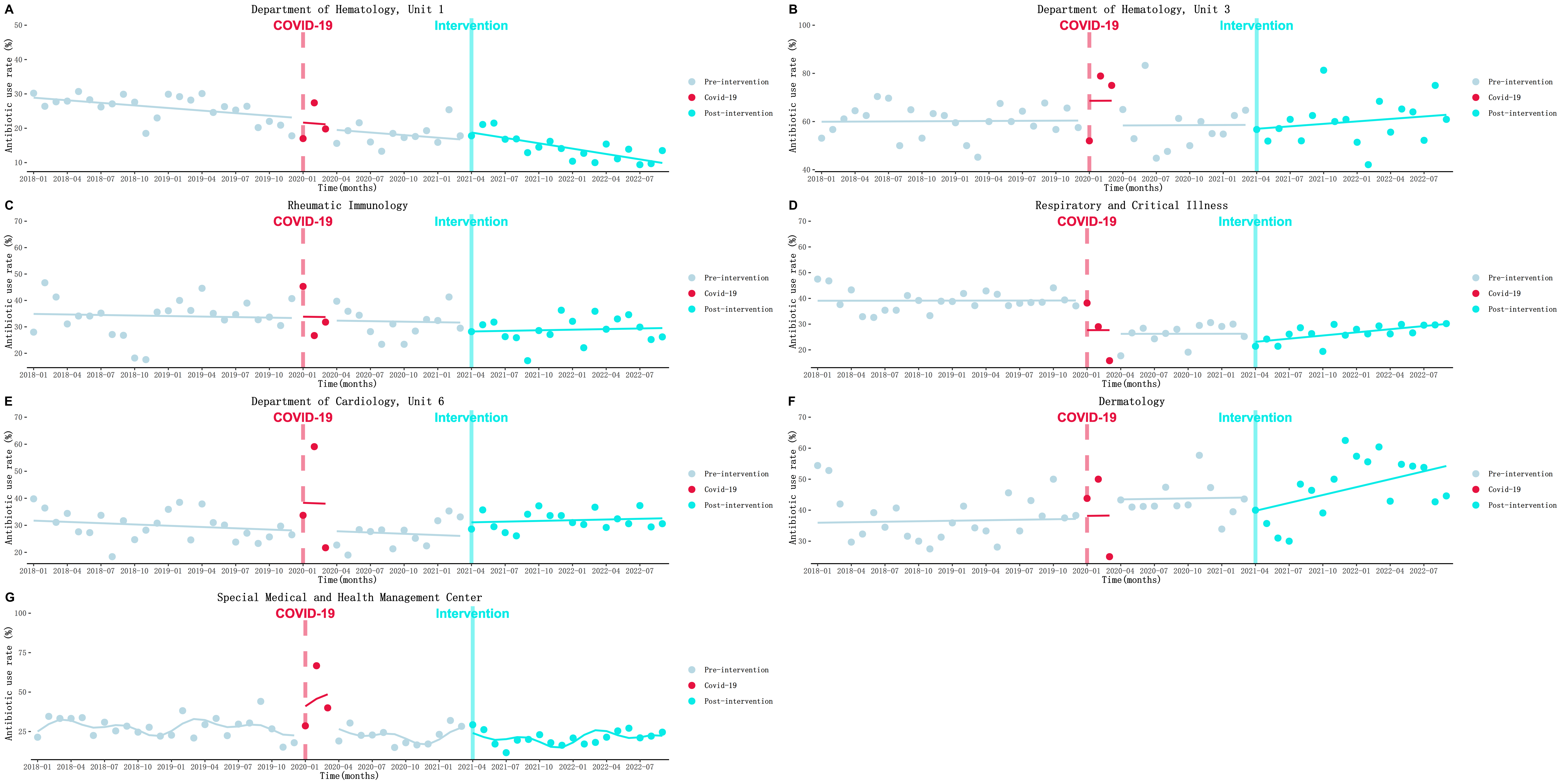


**Figure S4. Results of the ITS analysis of Internal Medicine system AUR**

**
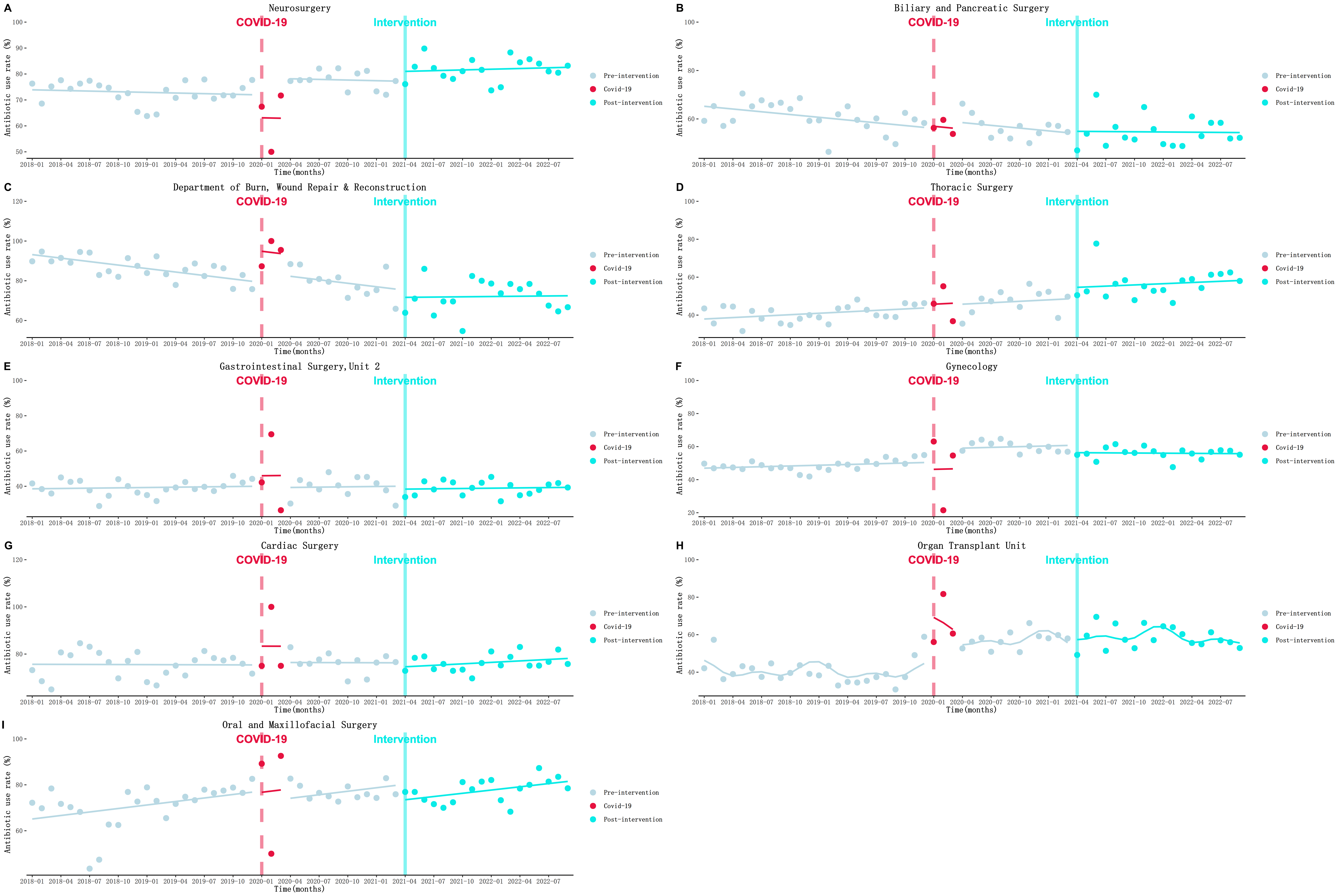
Figure S5. Results of the ITS analysis of Surgical system AUR**


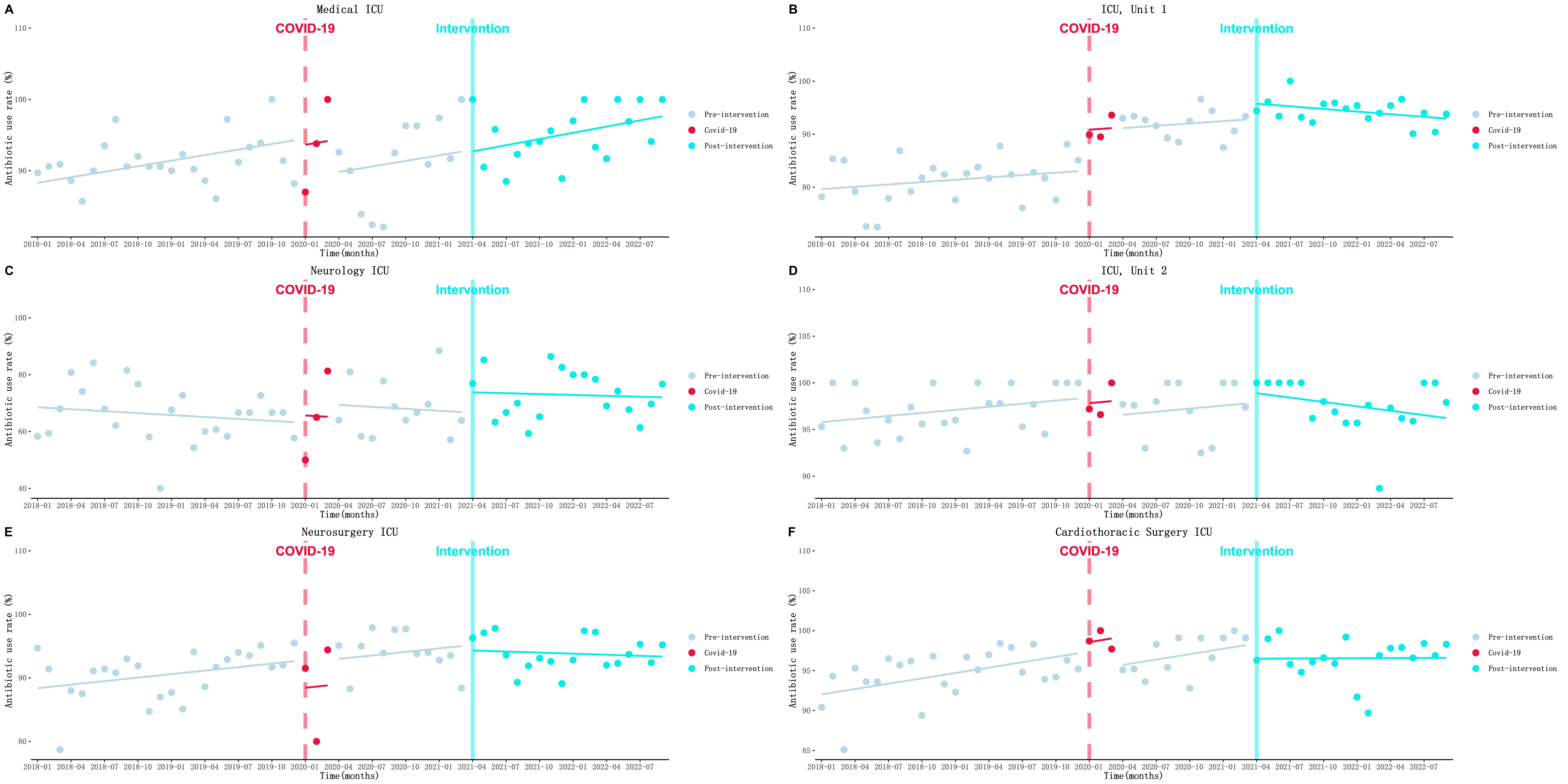


**Figure S6. Results of the ITS analysis of ICU system AUR**
